# Crystal Structure of a Novel Esterase Rv0045c from *Mycobacterium tuberculosis*

**DOI:** 10.1371/journal.pone.0020506

**Published:** 2011-05-26

**Authors:** Xiangdong Zheng, Jiubiao Guo, Lipeng Xu, Honglei Li, Dongwei Zhang, Kai Zhang, Fei Sun, Tingyi Wen, Siguo Liu, Hai Pang

**Affiliations:** 1 School of Medicine, Tsinghua University, Beijing, China; 2 Harbin Veterinary Research Institute, Chinese Academy of Agriculture, Harbin, China; 3 Department of Oral Biological and Medical Sciences, University of British Columbia, Vancouver, Canada; 4 Institute of Biophysics, Chinese Academy of Sciences, Beijing, China; 5 Department of Industrial Microbiology and Biotechnology, Institute of Microbiology, Chinese Academy of Sciences, Beijing, China; Institut de Pharmacologie et de Biologie Structurale, France

## Abstract

There are at least 250 enzymes in *Mycobacterium tuberculosis* (*M. tuberculosis*) involved in lipid metabolism. Some of the enzymes are required for bacterial survival and full virulence. The esterase Rv0045c shares little amino acid sequence similarity with other members of the esterase/lipase family. Here, we report the 3D structure of Rv0045c. Our studies demonstrated that Rv0045c is a novel member of α/β hydrolase fold family. The structure of esterase Rv0045c contains two distinct domains: the α/β fold domain and the cap domain. The active site of esterase Rv0045c is highly conserved and comprised of two residues: Ser154 and His309. We proposed that Rv0045c probably employs two kinds of enzymatic mechanisms when hydrolyzing C-O ester bonds within substrates. The structure provides insight into the hydrolysis mechanism of the C-O ester bond, and will be helpful in understanding the ester/lipid metabolism in *M. tuberculosis*.

## Introduction

*M. tuberculosis* is the most prevalent pathogen causing tuberculosis in humans and animals [1]. The bacteria is characterized by an unusual waxy coating on the cell surface (primarily mycolic acid) and it expresses more than 250 enzymes related to ester/lipid metabolism. In contrast, only about 50 enzymes are involved in the ester/lipid metabolism in *Escherichia coli* (*E. coli*) [2], [3]. These enzymes in *M. tuberculosis* which catalyze ester/lipid and carbohydrate metabolism are more likely to be required and essential to bacterial existence and survival [4]. In 2007, a cell wall-associated carboxyl esterase, Rv2224c, of *M. tuberculosis* H37Rv was identified as a major virulence gene and was further found to be required for bacterial survival in mice [5]. Rv0045c, participating in ester/lipid metabolism in *M. tuberculosis*, was predicted to be a hydrolase belonging to α/β hydrolase fold family based on bioinformatics studies. However, little is known about its substrate specificity and mechanism of action.

The α/β hydrolase fold was identified in 1992, by comparing five hydrolytic enzymes with widely different catalytic function [6]. Since then, more than 50 members belonging to this family have been identified and characterized by structure determination [7]. The α/β hydrolase fold involves a variety of enzymes including esterases, lipases, epoxide hydrolases, dehalogenases, proteases, and peroxidases, making it one of the most versatile protein families known [8]. The conversed feature of the α/β hydrolase fold has been described as a mostly parallel, eight-stranded β sheet surrounded on both sides by α helices (only the second β strand is antiparallel) [9]–[11].

The Rv0045c gene encodes a polypeptide chain of 298 amino acids with a putative hydrolase activity. Sequence comparisons show that Rv0045c shares a low sequence identity (<30%) to other members of the α/β hydrolase fold family, however, the consensus sequence G-X-S-X-G of the nucleophile elbow and the catalytic residues are highly conserved. Similar to other α/β hydrolases, it has been previously shown that Rv0045c can hydrolyze ester bonds within a series of *p*-nitrophenyl derivatives (C_2_–C_14_) [12]. The purified enzyme can effectively hydrolyze *p*-nitrophenyl derivatives with short hydrocarbon chains, especially C_2_–C_8_. We identified *p*-nitrophenyl caproate (C_6_) as the most suitable substrate of Rv0045c at the assay conditions of 39°C and pH 8.0 [12].

To understand the active site and enzymatic mechanism of esterase Rv0045c, we determined the crystal structure of the enzyme and performed docking experiments. Our studies clearly revealed that 1) Rv0045c contains two distinguished domains: the α/β fold domain and the cap domain, 2) Rv0045c, from *M. tuberculosis*, is a novel member of α/β hydrolase fold family, and 3) Rv0045c probably employs two kinds of enzymatic mechanisms (indirect and/or direct) where S154 attacks the carbonyl carbon within the C-O ester bond using or without using an activated water molecule.

## Results

### Structure determination and features of Rv0045c

**Figure 1:**
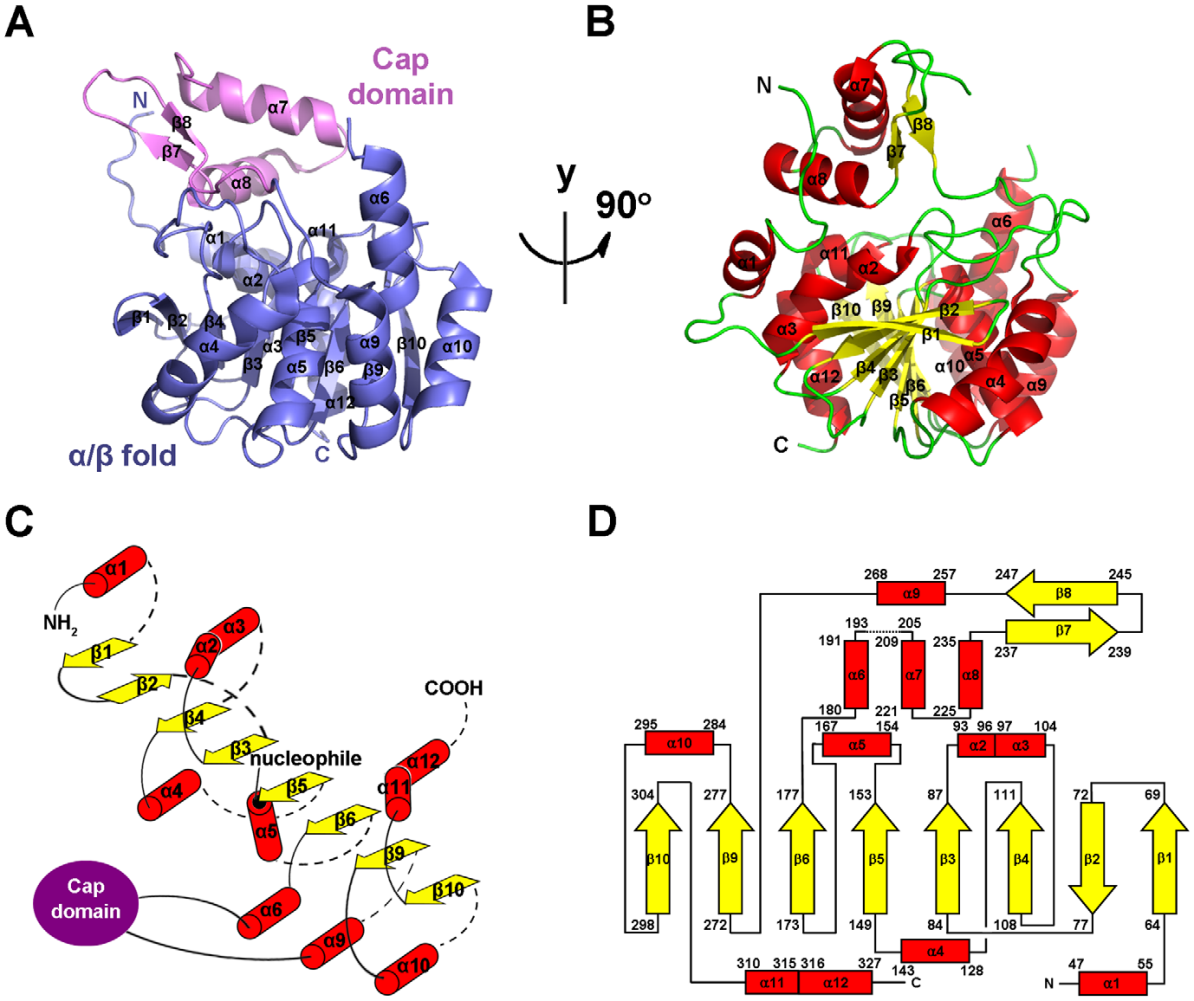
Overall structure of Rv0045c. (A–B) Cartoon representation of Rv0045c in two views related by a vertical rotation of 90 degrees. The secondary structural elements (α1–α12, β1–β10) were labeled. The core structural elements are colored slate and the cap domain in violet (A). α-helices (red) and β-strands (yellow) are differentiated by colors (B). (C) Secondary structure diagram of the α/β fold core of Rv0045c. α-helices, β-strands and the cap domain are represented by red cylinders, yellow arrows and a violet ellipse, respectively. The α/β fold core consists of a mostly parallel, 8-stranded β sheet surrounded on both sides by α-helices (only β2 is antiparallel). The nucleophilic residue, Ser154, positioned at the beginning of α5, is marked with a black dot. (D) Topology diagram of Rv0045c using the same color scheme as (B). The missing region between α6 and α7 (residues 194–204) is represented as dotted line.

The purified Rv0045c protein and selenomethionine (Se-Met) labeled Rv0045c protein were crystallized in the same condition (0.2 M MgCl_2_, 100 mM imidazole, pH 7.0, 19% (*w*/*v*) PEG 4000). However, both crystals revealed different space groups (Table 1). The crystal structure of Rv0045c was determined by SAD and was refined to 2.8 Å resolution. The final model (Fig. 1) of Rv0045c consists of residues 38–193 and 205–329 with the missing residues being not visible in the density maps. The analysis of Ramachandran plot by COOT [13] showed that most of the modeled residues were in preferred and allowed regions (Table 1). The model clearly contains two distinct structural domains: an almost globular α/β fold domain (α1β1β2β3α2α3β4α4β5α5β6α6α9β9α10β10α11α12) and an inserted cap domain (α7α8β7β8) which interacts with the α/β fold domain.

**Table 1:** Data collection and refinement statistics.

	Native Rv0045c	SeMet Rv0045c
**Data Collection**		
Synchrotron	SSRF	PF-BL17A
Wavelength (Å)	1.072	0.9790
Resolution (Å)	50-2.8	50-2.6
Space group	P3_1_	P3_1_21
Cell-unit parameters (Å)	a = b = 73.465, c = 48.063	a = b = 130.330, c = 48.785
Matthew's coefficient	2.1	3.4
% solvent	41.7	64.0
No. of molecule per ASU	1	1
No. observations	47965	157440
No. unique reflections	7159	14797
Redundancy	6.7 (5.3)	10.6 (8.3)
R_sym_ a	0.079 (0.397)	0.144 (0.778)
Mean *I*/σ*_I_*	26.5 (3.0)	17.7 (2.0)
Completeness (%)	100.0 (100.0)	100.0 (99.7)
**Refinement**		
Resolution (Å)	38.35-2.8	
No. reflections (total)	6810	
No. reflections (free)	336	
R factor (%)^b^	21.69	
R_free_ (%)^b^	28.57	
Figure of merit	0.8296	
No. of waters	36	
Overall B factor	53.10	
Wilson B factor	80.80	
rmsd bond lengths (Å)^c^	0.0129	
rmsd bond angles (^O^)^c^	1.570	
Ramachandran plot		
Preferred (%)	93.5	
Allowed (%)	6.1	
Outliers (%)	0.4	

a R_sym_ = Σ*_h_*Σ*_i_* | *I_h,I_*−*I_h_* |/Σ*_h_*Σ*_i_I_h,i_*, where *I_h_* is the mean intensity of the *i* observations of symmetry-related reflections of *h*.

b R = Σ| *F*_obs_−*F*_calc_ |/Σ*F*_obs_, where *F*_obs_ = *F*_P_, and *F*_calc_ is the calculated protein structure factor from the atomic model (R_free_ was calculated with 5% of the observed reflections).

c rmsd (root-mean-square deviation) in bond lengths and angles are the deviations from ideal values.

### Structure of the α/β fold domain

**Figure 2:**
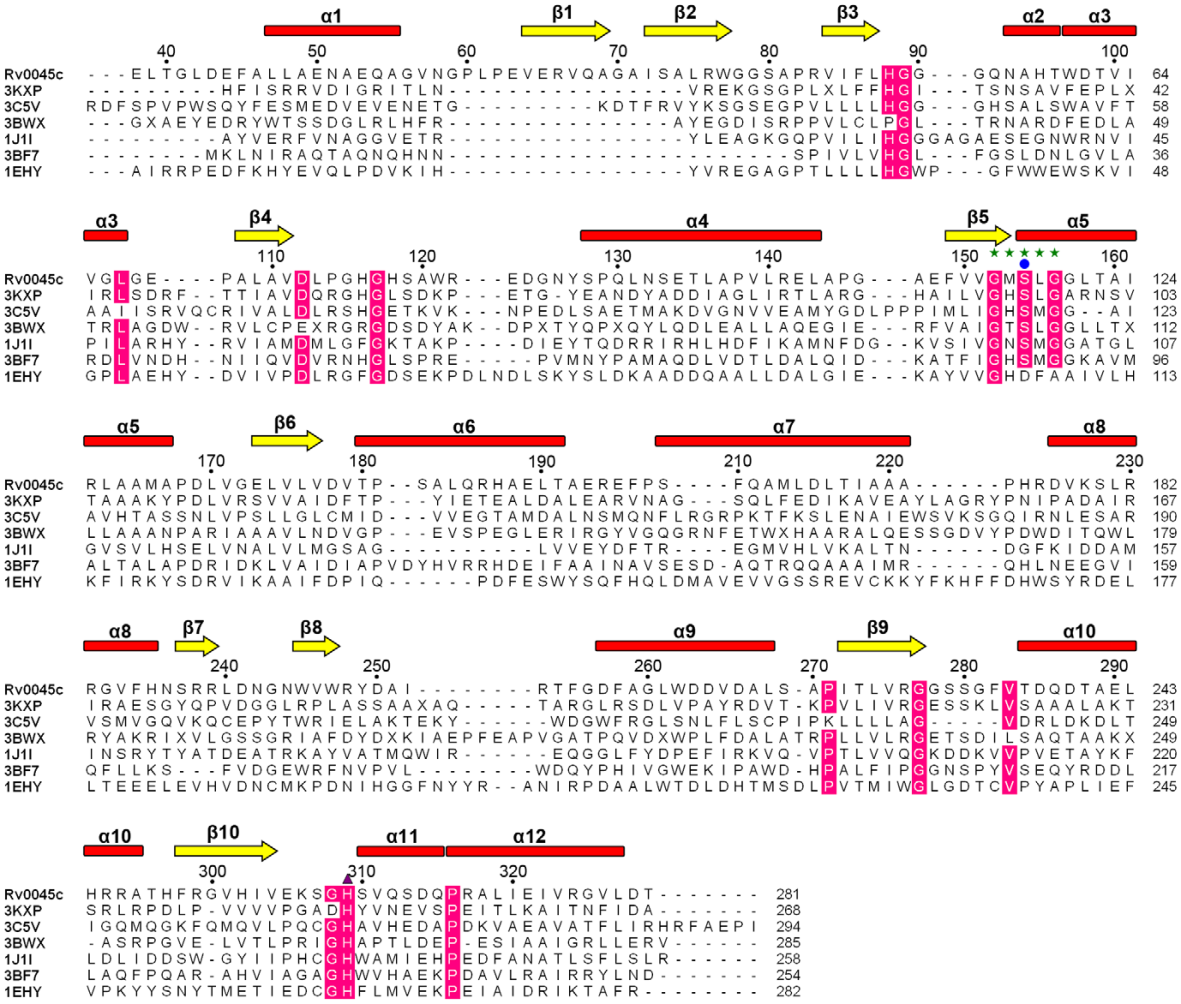
Sequence alignment of Rv0045c with other structurally homologous enzymes. Included enzymes are E-2AMS hydrolase (PDB ID: 3KXP), methylesterase PME-1 (PDB ID: 3C5V), hydrolase YP_496220.1 (PDB ID: 3BWX), CarC enzyme (PDB ID: 1J1I), esterase ybfF (PDB ID: 3BF7) and soluble epoxide hydrolase (PDB ID: 1EHY). Secondary structural elements and every the tenth residue of Rv0045c are indicated above the alignment. The “nucleophile elbow” of G-X-S-X-G sequence motif is marked with green stars and the nucleophilic serine residue Ser with a blue circle. The other catalytic residue is labeled using a purple triangle. Strictly conserved residues with the identity of >80% are highlighted by pink front.

Like other members of the α/β hydrolase fold family, the α/β fold domain represents the core of Rv0045c (Fig. 1A and 1B). The α/β fold domain of Rv0045c consists of a mostly parallel, 8-stranded β sheet surrounded by α-helices on both sides (only the second strand is antiparallel), which has been regarded as the “canonical” feature of the α/β hydrolase fold family [8]. The last strand is oriented with a twisting angle of approximately 120° to the first one (Fig. 1B). The topology of β-α-β motifs (β3-α2-α3-β4, β5-α5-β6 and β9-α10-β10) in the centre displays a right-handed super helical twist (Fig. 1C). The α/β hydrolase fold domain provides the stable scaffold for the active site of Rv0045c. Sequence alignments revealed that the “nucleophile elbow” of G-X-S-X-G sequence motif is located in the sharp turn connecting β5 and α5 (Fig. 1C and 2) and is highly conversed among these enzymes (Fig. 2), although Rv0045c shows no significant sequence homology to any other α/β hydrolase fold family member.

### Structure of the cap domain

**Figure 3:**
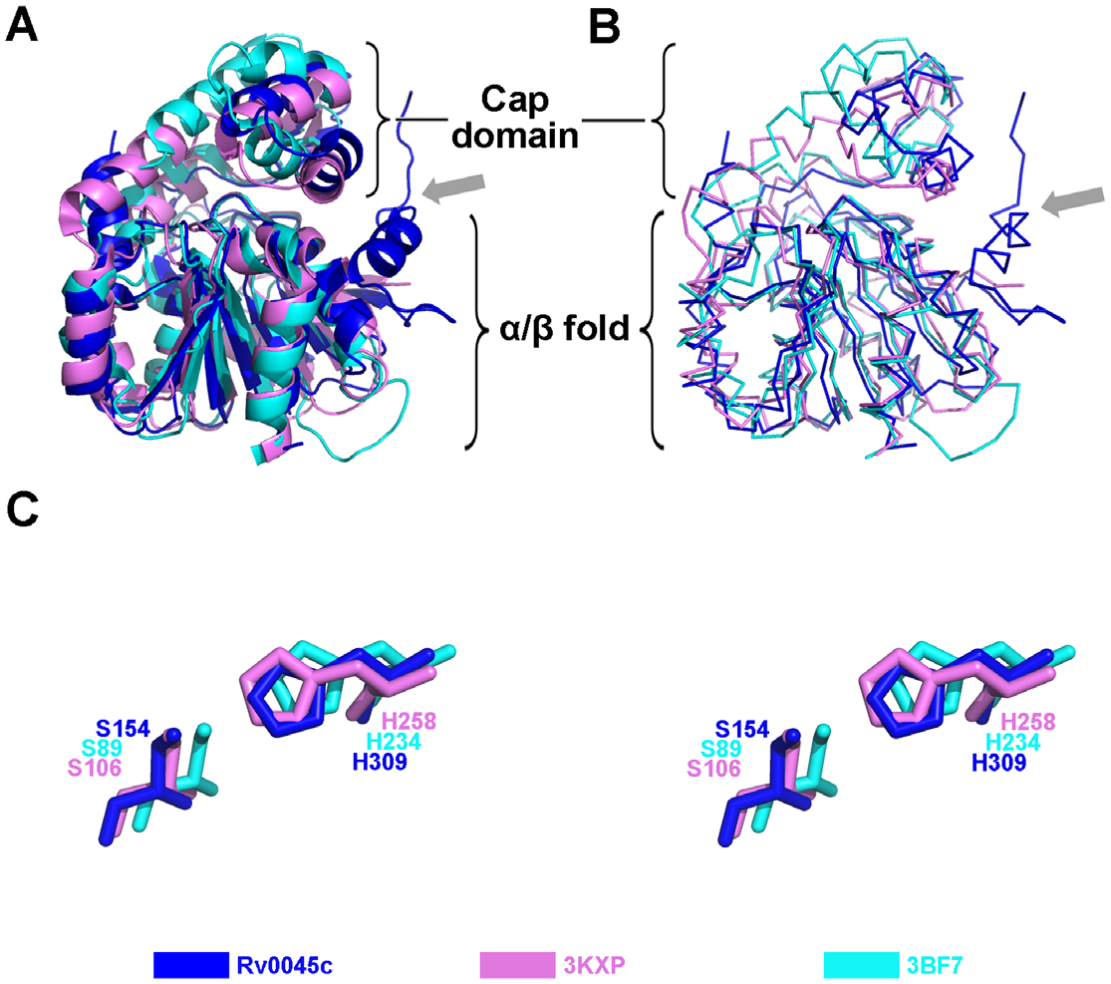
Superposition of Rv0045c with other members of the α/β hydrolase fold family. Cartoon (A) and ribbon (B) diagrams are presented. The α/β fold core of Rv0045c (blue) superposes well with those of E-2AMS hydrolase from *Mesorhizobium loti* (PDB ID: 3KXP) (violet) and esterase ybfF from *Escherichia coli* (PDB ID: 3BF7) (cyan) except for α1 which is marked with a gray arrow. (C) Close-up view of catalytic residues. The catalytic residues of three α/β fold cores are differentiated and labeled using the same color scheme as (A) and (B).

**Figure 4:**
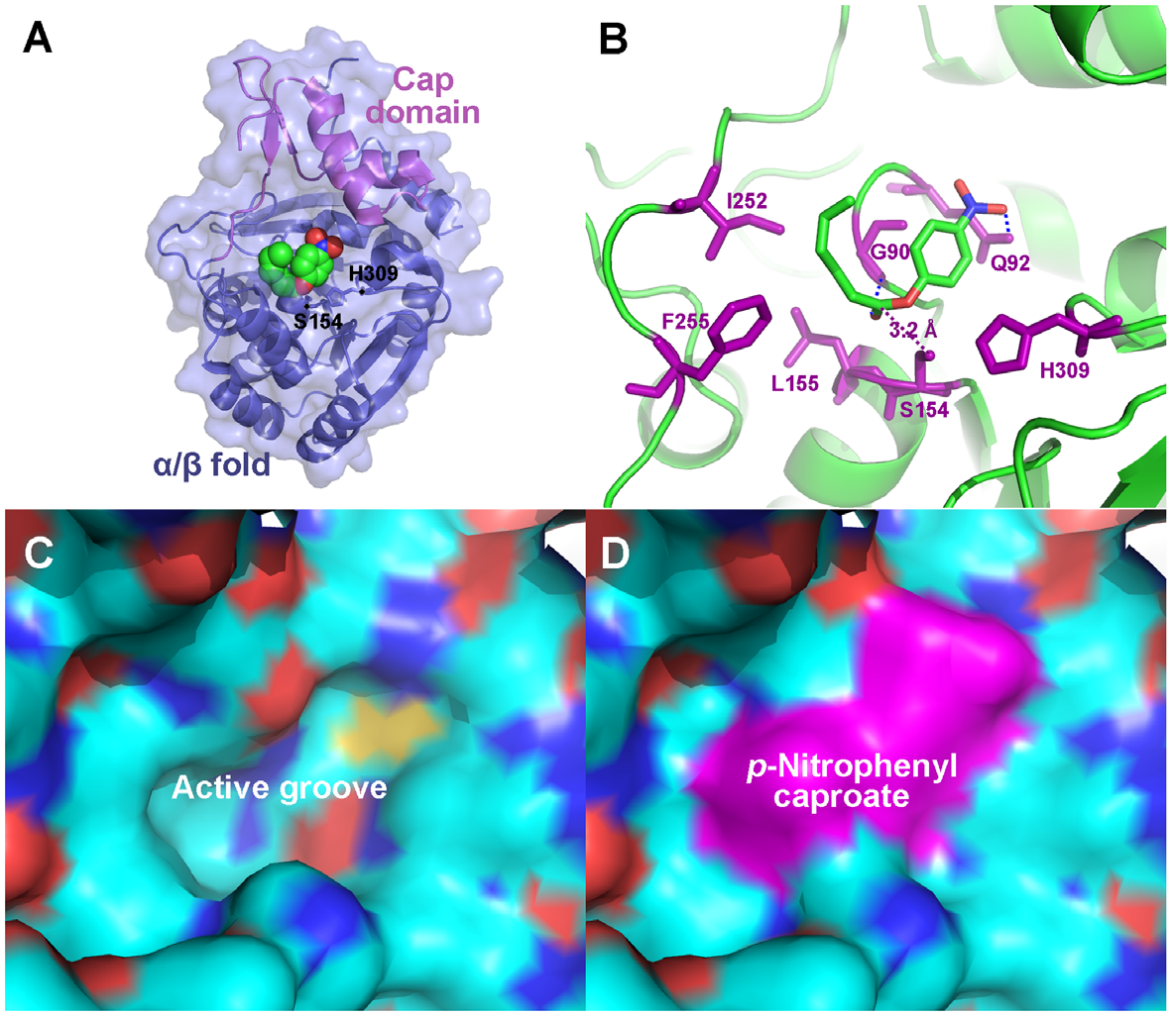
Modeling of *p*-nitrophenyl caproate in the active site of Rv0045c. (A) The modeled *p*-nitrophenyl caproate, which is displayed as space-filling pattern, was bound to the active site under the cap domain. The cap domain and α/β fold of Rv0045c are differentiated by colors. Ser154 and His309 within the active site are labeled. (B) Ribbon diagram of the binding site and active site of Rv0045c with *p*-nitrophenyl caproate manually modeled. The binding site is formed by Gly90, Gln92, Leu155, Ile252 and Phe255. The compound was stabilized by two hydrogen bound (shown as blue dotted line) contributed by Gly90 and Gln92. The binding site and active site constitute the active groove (C) on the surface of Rv0045c and the modeled *p*-nitrophenyl caproate fits it quite well (D).

The polypeptide region, Arg205 - Ile252, in Rv0045c forms the cap domain. The cap domain comprises two sequential α-helices (α7, α8) and another two consecutive β-strands (β7, β8). Unlike the α/β fold domain, structural homologies of the cap domain cannot be absolutely identified among the superposed α/β hydrolase fold family members (Fig. 3A and 3B). The alignment and orientation of α-helices and β-strands within the cap domain show a little difference. The inserted cap domain is supposedly related to substrate binding both in E-2AMS hydrolase [14] and esterase ybfF [15] and may provide clues about these two enzymes' substrate specificity, however, no devotion contributed by the cap domain of Rv0045c was revealed when *p*-nitrophenyl caproate was docked into the active site (Fig. 4A and 4B). Residues 194–204 are missed in this domain, for the reason that this region is much more flexible and reveals very poor electron density.

### Active site of Rv0045c

The putative active site of Rv0045c was identified via sequence alignment (Fig. 2) and structural homology (Fig. 3) with other α/β fold hydrolases. The active site formed by Ser154 and His309 is shielded by the cap domain. The putative nucleophilic residue Ser154 is located at the beginning of α5. Resultsof docking experiment indicated that Gly90, Gln92, Leu155, Ile252 and Phe255 help *p*-nitrophenyl caproate locate onto the active site, and that these residues comprise the binding site of Rv0045c (Fig. 4B). Three hydrophobic residues, including Leu155, Ile252 and Phe255, contribute to the stable conformation of the hydrocarbon chain of *p*-nitrophenyl caproate. The substrate is further stabilized by two hydrogen bond contributed by Gly90 and Gln92 (Fig. 4B, blue dotted line). Residues involved in forming active site and binding site devote themselves shaping the active groove (Fig. 4C), which can well accomodate *p*-nitrophenyl caproate (Fig. 4D).

## Discussion

The α/β hydrolase fold family has been structurally well characterized and comprises a variety of enzymes including esterases, lipases, epoxide hydrolases, dehalogenases, proteases, and peroxidases, catalyzing myriad reactions. Analysis of the primary sequence for Rv0045c using BLAST suggested that this enzyme shares little sequence identity to other members of the α/β hydrolase fold family, though the enzyme was structurally characterized to be a novel member of the family. A DALI [16] search was performed using the structure of Rv0045c, and these results confirmed that Rv0045c shows little sequence identity but high structural similarity to other members of the α/β hydrolase fold family. Related members of this family are shown in Table 2, and their similarity to Rv0045c is presented by Z score, rmsd, identity and number of aligned residues [14], [15], [17]–[19]. Data of superposition of Rv0045c with E-2AMS hydrolase and esterase ybfF showed that the cores of the three enzymes, which are all comprised of eight stranded β-sheets with α-helices on both sides, overlap with each other (Fig. 3A and 3B). However, there is a little difference in the alignment and orientation of the cap domain. The cap domain of Rv0045c is an insertion between α6 and α9, including two stranded antiparallel β-sheets (β7 and β8) and two α-helices (α7 and α8). This feature is similar to that in E-2AMS hydrolase and esterase ybfF, but the cavity formed by the cap domain and the α/β fold domain in esterase ybfF is a little more expanding. The flexible region in the cap domain, from residue 194 to 204, is not visible in the density map of Rv0045c. The cap domain and the α/β fold domain within Rv0045c provide a wide and open cavity for larger substrates. It is probably that the missing region becomes to be stable and visible when a large substrate is bond to the protein. In that case, the interaction between the cap domain and substrate may transform the flexible region into a stable conformation.

**Table 2:** Enzymes Identified as Structurally Homologous to Rv0045c through DALI.

	PDB ID	Z score	rmsd (Å)	Identity (%)	NRES^a^	RN^b^
E-2AMS hydrolase	3KXP	28.3	2.3	24	268	14
Methylesterase PME-1	3C5V	24.3	2.9	20	294	17
hydrolase YP_496220.1	3BWX	24.3	3.2	21	285	
CarC enzyme	1J1I	23.8	2.7	18	258	18
esterase ybfF	3BF7	23.1	3.0	20	254	15
soluble epoxide hydrolase	1EHY	23.0	3.0	17	282	19

a NRES = No. of aligned residues.

b RN = Reference Number.

The members of α/β hydrolase fold family utilize a highly conserved catalytic nucleophile which contains a serine, cysteine or aspartic acid residue [8]. The nucleophile of Rv0045c is Ser154, positioned as the first residue at the beginning of α5. The active site of Rv0045c (Ser154 and His309) identified by sequence alignment is highly conserved among the enzymes aligned. Both in E-2AMS hydrolase and esterase ybfF, the cap domain directly contributes to the substrate binding, which is not observed in Rv0045c when *p*-nitrophenyl caproate was docked into the active site. As shown in a docking experiment, when a small substrate, *p*-nitrophenyl caproate, was bound, the cap domain is not involved in the binding of the substrate to the protein. The binding site is located on the surface of the α/β fold domain. Three hydrophobic residues (Leu155 in α5, Ile252 and Phe255 after β8) help the hydrocarbon chain of *p*-nitrophenyl caproate to obtain an optimum conformation to reduce the binding energy. The orientation of the C-O ester bond of *p*-nitrophenyl caproate is stabilized via two hydrogen bonds contributed by Gly90 and Gln92 after β3. It has been already known that Ser is an executive residue in both E-2AMS hydrolase and esterase ybfF [14], [15]. To confirm the activity of Ser154 within Rv0045c, we generated a mutant of this enzyme, but no any activity could be detected (data not shown).

**Figure 5:**
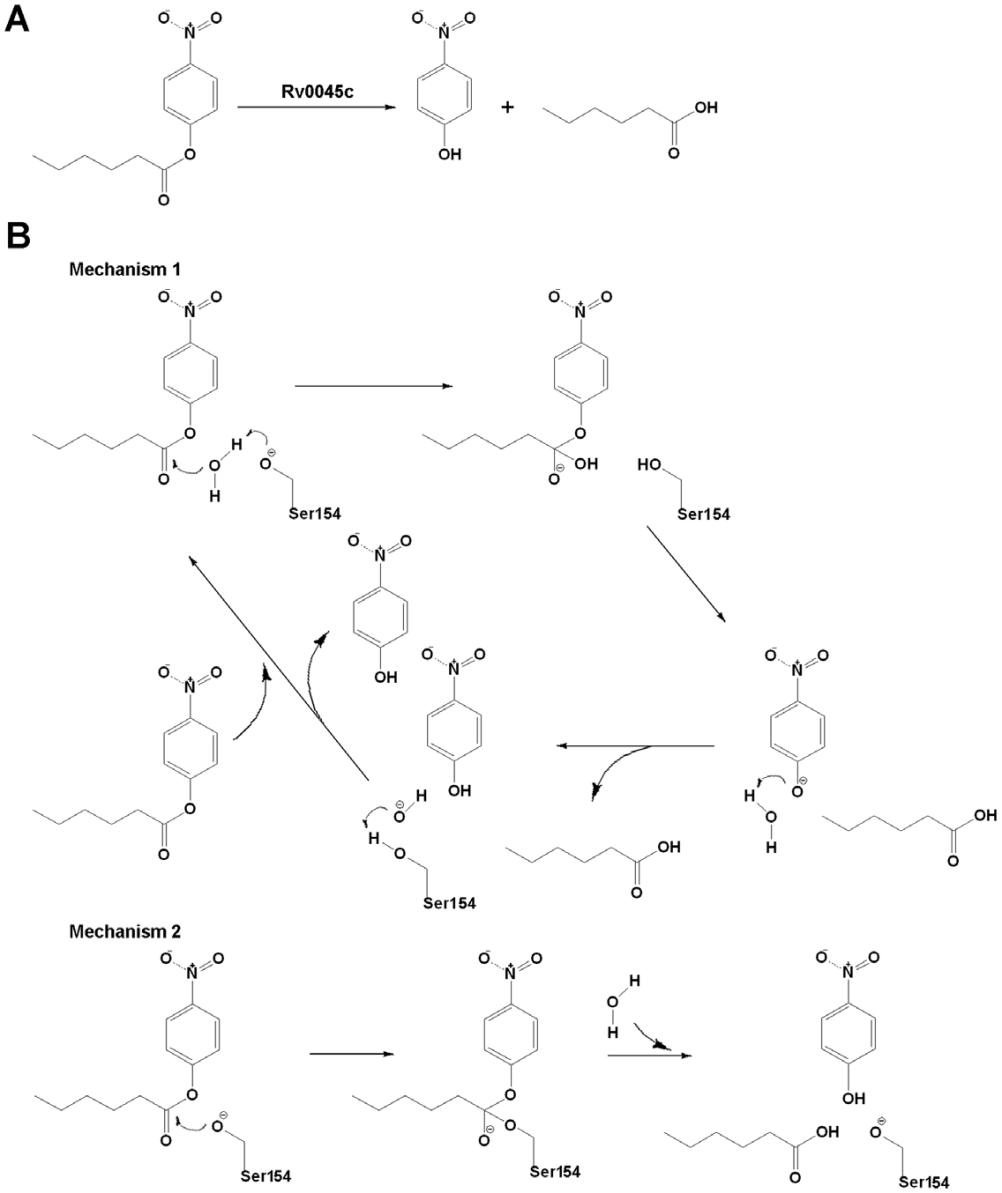
Proposed mechanisms for the hydrolysis of *p*-nitrophenyl caproate. (A) The reaction for the hydrolysis of *p*-nitrophenyl caproate by Rv0045c. Rv0045c hydrolyzes *p*-nitrophenyl caproate to produce *p*-nitrophenol and caproic acid. (B) Proposed mechanisms for the hydrolysis reaction. Mechanism 1 utilizes Ser154 to activate a water molecule for attacking the carbonyl carbon of the C-O ester bond. Mechanism 2 utilizes Ser154 to directly attack the carbonyl carbon of the C-O ester bond.

A previous study about the biochemical activity of Rv0045c suggested that the enzyme can hydrolyze the ester bond of *p*-nitrophenyl derivatives and *p*-nitrophenyl caproate was identified as the most effective substrate [12]. As an esterase, Rv0045c can hydrolyze the C-O ester bond of *p*-nitrophenyl caproate to produce *p*-nitrophenol and caproic acid (Fig. 5A). In the model of Rv0045c binding *p*-nitrophenyl caproate, the hydroxyl oxygen of Ser154 is 3.2 Å (purple dotted line, Fig. 4B) from the carbonyl carbon of the C-O ester bond of the substrate. The indirect and direct enzymatic mechanisms of Rv0045c can be subsequently hypothesized (Fig. 5B). It is probable that Ser154 interacts with the C-O ester bond indirectly, using an activated water molecule (Mechanism 1, Fig. 5B), for the reason that it is too long (3.2 Å) for Ser154 to directly attack the carbonyl carbon within the C-O ester bond. Similar to the mechanism proposed in the model of E-2AMS hydrolase [14], there must be some small molecules, for instance the water molecules, mediating the hydrolysis reaction. In detail, the hydroxyl oxygen of Ser154 is firstly polarized by adjacent His309 before Ser154 attacks the hydrogen atom of a free water molecule, and then, the activated water molecule attacks the carbonyl carbon within the C-O ester bond.

However, it cannot be ignored that the binding of substrate to Rv0045c may cause conformational change of the enzyme. In that case, Ser154 might be close enough to directly attack the carbonyl carbon within the C-O ester bond and the enzyme employed a direct mechanism (Mechanism 2, Fig. 5B). Rv0045c can catalyze a mount of substrates with hydrocarbon chains of different length. We infer that Rv0045c may adopt different enzymatic mechanisms (direct and/or indirect) when binding different substrates. We have performed co-crystallization with ligands, however, no esterase Rv0045c-substrate complex has been successfully crystallized by now. We will continue to seek the way to get solvable crystals of Rv004c-substrate complex to clarify the catalytic mechanism of Rv0045c.

Tuberculosis is a contagious respiratory system disease, which is caused by *M. tuberculosis* via infecting the lungs of mammalian. *M. tuberculosis* can tolerate and withstand rigorous condition and weak disinfectants to survive in a dry state for weeks. It was reported that the unusual cell wall, rich in lipids, is likely responsible for this resistance [20]. Rv0045c is proposed to be an esterase or hydrolase involved in lipid metabolism. Our study determines for the first time the structure of Rv0045c and will give further insight into the mechanism of esters or lipids hydrolysis in *M. tuberculosis*. This work will help to design and screen inhibitors against Rv0045c to verify the function and role of this enzyme in *M. tuberculosis*.

## Materials and Methods

### Protein preparation

The expression construct was generated using a standard PCR procedure. Full-length Rv0045c was sub-cloned into pET28a vector (Invitrogen). The production induced with 0.3 mM IPTG was overexpressed at 16°C for 20 h in *E. coli* BL21 (DE3) strain (Novagen). The soluble fraction of Rv0045c from cell lysate was purified by Ni Sepharose™ 6 Fast Flow resin (GE Healthcare) to homogeneity and further polished by ion-exchange chromatography (Resource Q and S 1 mL, GE Healthcare) and gel filter chromatography (Superdex 75 10/300 GL, GE Healthcare). Se-Met labeled Rv0045c was produced by growing the *E. coli* cells in a minimum medium containing selenomethionine and purified in the same way as described above.

### Crystallization and data collection

The diffracting crystals of native and Se-Met labeled Rv0045c were grown at 16°C using the hanging-drop vapor-diffusion method by mixing 1 µL protein (5 mg/mL) with an equal volume of reservoir solution. The Crystal Screen kit I and Crystal Screen kit II of Hampton Research (Aliso Viejo, CA, USA) were used for preliminary screen. Both the native and Se-Met labeled Rv0045c were crystallized in the same condition consisting of 0.2 M MgCl_2_, 100 mM Tris-HCl pH 8.5, 30% (*w/v*) PEG4000 with, however, the different space groups. The native crystals are in the space group P3_1_, with unit cell parameters a = b = 73.465 Å, c = 48.063 Å, and the Se-Met labeled crystals in P3_1_21 with a = b = 130.330 Å, c = 48.785 Å. For data collection, 20% (v/v) glycerol was added to the crystallizing precipitant as a cryoprotectant and the crystals were flash frozen in a −173°C nitrogen-gas stream. A complete 2.8 Å native dataset and a complete 2.6 Å Se-Met MAD dataset were respectively collected on beamline BL17U at Shanghai Synchrotron Radiation Facility (SSRF, Shanghai, China) and beamline BL17A at the Photon Factory (Tsukuba, Japan) and processed using the HKL-2000 program package [21].

### Structure determination

The structure of Rv0045c was determined by single-wavelength anomalous dispersion (SAD). Selenium atom coordinates were determined using the HKL2MAP [22] program suite and initial SAD phases were calculated and improved with the program SOLVE/RESOLVE [23], [24]. The residues of Rv0045c were built manually using the program COOT [13] and the refinement was performed with CCP4 refmac5 [25]. The Rv0045c crystal structure has been refined to 2.8 Å resolution and working and free R factors are 21.69% and 28.57%, respectively. The PyMOL (http://www.pymol.org) molecular graphics program of DeLano Scientifics was used to present the final structure and to produce figures. The data statistics are summarized in Table 1.

### Docking experiment

For docking experiment, the AutoDockTool [26]–[28] software was used for macromolecule and ligand preparing, macromolecule-ligand docking and result analysis. The orientations of nitro-group and hydrocarbon chain of *p*-nitrophenyl caproate were allowed to rotate until the favorable docking position and conformation were found. The docking did not require reorientation of the macromolecule side chains.

### Accession numbers

The atomic coordinates and structure factors of Rv0045c (PDB ID: 3P2M) have been deposited in the Protein Data Bank (www.pdb.org).

## References

[1] Ryan KJ, Ray CG (2004). Sherris Medical Microbiology (4th Ed.).

[2] Cole ST, Brosch R, Parkhill J, Garnier T, Churcher C (1998). Deciphering the biology of *Mycobacterium tuberculosis* from the complete genome sequence.. Nature.

[3] Camus JC, Pryor MJ, Médigue C, Cole ST (2002). Re-annotation of the genome sequence of *Mycobacterium tuberculosis* H37Rv.. Microbiology.

[4] Sassetti CM, Rubin EJ (2003). Genetic requirements for mycobacterial survival during infection.. Proc Natl Acad Sci U S A.

[5] Lun S, Bishai WR (2007). Characterization of a novel cell wall-anchored protein with carboxylesterase activity required for virulence in *Mycobacterium tuberculosis*.. J Biol Chem.

[6] Ollis DL, Cheah E, Cygler M, Dijkstra B, Frolow F (1992). The alpha/beta hydrolase fold.. Protein Eng.

[7] Heikinheimo P, Goldman A, Jeffries C, Ollis DL (1999). Of barn owls and bankers: a lush variety of alpha/beta hydrolases.. Structure.

[8] Nardini M, Dijkstra BW (1999). Alpha/beta hydrolase fold enzymes: the family keeps growing.. Curr Opin Struct Biol.

[9] Hubbard TJ, Ailey B, Brenner SE, Murzin AG, Chothia C (1998). SCOP, Structural Classification of Proteins database: applications to evaluation of the effectiveness of sequence alignment methods and statistics of protein structural data.. Acta Crystallogr D Biol Crystallogr.

[10] Orengo CA, Martin AM, Hutchinson G, Jones S, Jones DT (1998). Classifying a protein in the CATH database of domain structures.. Acta Crystallogr D Biol Crystallogr.

[11] Cousin X, Hotelier T, Giles K, Toutant JP, Chatonnet A (1998). aCHEdb: the database system for ESTHER, the alpha/beta fold family of proteins and the Cholinesterase gene server.. Nucleic Acids Res.

[12] Guo JB, Zheng XD, Xu LP, Liu ZY, Xu KH (2010). Characterization of a Novel Esterase Rv0045c from *Mycobacterium tuberculosis*.. PLoS ONE.

[13] Emsley P, Cowtan K (2004). Coot: model-building tools for molecular graphics.. Acta Crystallogr D Biol Crystallogr.

[14] McCulloch KM, Mukherjee T, Begley TP, Ealick SE (2010). Structure determination and characterization of the vitamin B6 degradative enzyme (E)-2-(acetamidomethylene)succinate hydrolase.. Biochemistry.

[15] Park SY, Lee SH, Lee J, Nishi K, Kim YS (2008). High-resolution structure of ybfF from *Escherichia coli* K12: a unique substrate-binding crevice generated by domain arrangement.. J Mol Biol.

[16] Holm L, Rosenström P (2010). Dali server: conservation mapping in 3D.. Nucleic Acids Res.

[17] Xing Y, Li Z, Chen Y, Stock JB, Jeffrey PD (2008). Structural mechanism of demethylation and inactivation of protein phosphatase 2A.. Cell.

[18] Habe H, Morii K, Fushinobu S, Nam JW, Ayabe Y (2003). Crystal structure of a histidine-tagged serine hydrolase involved in the carbazole degradation (CarC enzyme).. Biochem Biophys Res Commun.

[19] Nardini M, Ridder IS, Rozeboom HJ, Kalk KH, Rink R (1999). The x-ray structure of epoxide hydrolase from *Agrobacterium radiobacter* AD1. An enzyme to detoxify harmful epoxides.. J Biol Chem.

[20] Murray PR, Rosenthal KS, Pfaller MA (2005). Medical Microbiology (5th Ed.).

[21] Otwinowski Z, Minor W (1997). Processing of X-ray diffraction data collected in oscillation mode.. Method Enzymol.

[22] Pape T, Schneider TR (2004). *HKL2MAP*: a graphical user interface for macromolecular phasing with *SHELX* programs.. J Appl Crystallogr.

[23] Terwilliger TC (2002). Automated structure solution, density modification and model building.. Acta Crystallogr D Biol Crystallogr.

[24] Terwilliger T (2004). SOLVE and RESOLVE: automated structure solution, density modification and model building.. J Synchrotron Radiat.

[25] Murshudov GN, Vagin AA, Dodson EJ (1997). Refinement of macromolecular structures by the maximum-likelihood method.. Acta Crystallogr D Biol Crystallogr.

[26] Goodsell DS, Olson AJ (1990). Automated docking of substrates to proteins by simulated annealing.. Proteins.

[27] Morris GM, Goodsell DS, Huey R, Olson AJ (1996). Distributed automated docking of flexible ligands to proteins: parallel applications of AutoDock 2.4.. J Comput Aided Mol Des.

[28] Morris GM, Goodsell DS, Halliday RS, Huey R, Hart WE (1998). Automated docking using a Lamarckian genetic algorithm and an empirical binding free energy function.. J Comput Chem.

